# Preoperative Endoscopic Ultrasound Strain Elastography Has Limited Value for Predicting Intraoperative Pancreatic Texture: A Single‐Operator Pilot Study

**DOI:** 10.1002/jgh3.70304

**Published:** 2025-11-16

**Authors:** Kengo Matsumoto, Akira Doi, Shiro Hayashi, Masashi Yamamoto, Koji Fukui, Masafumi Yamashita, Junzo Shimizu, Tsutomu Nishida

**Affiliations:** ^1^ Department of Gastroenterology Toyonaka Municipal Hospital Toyonaka Osaka Japan; ^2^ Department of Gastroenterology and Internal Medicine Hayashi Clinic Suita Osaka Japan; ^3^ Department of Gastroenterological Surgery Toyonaka Municipal Hospital Toyonaka Osaka Japan

**Keywords:** elasticity imaging techniques, endoscopic ultrasound, palpation, pancreas/diagnostic imaging, strain elastography

## Abstract

**Background/Aims:**

Soft pancreatic texture is a key risk factor for postoperative pancreatic fistula (POPF). However, preoperative quantification remains difficult. Intraoperative palpation is the current standard for assessing pancreatic hardness; however, this technique is not feasible in robot‐assisted surgery. We prospectively evaluated whether endoscopic ultrasound strain elastography (EUS‐SE) could predict pancreatic hardness.

**Methods:**

Nine consecutive patients scheduled for pancreatectomies underwent preoperative EUS‐SE. The strain ratio (SR) values from the nontumorous parenchyma were compared using intraoperative palpation (soft vs. hard). A receiver operating characteristic (ROC) analysis was used to determine the optimal SR cutoff. The associations between computed tomography (CT) attenuation, fibrosis, and postoperative outcomes were examined.

**Results:**

Six pancreases were classified as soft, and three pancreases were classified as hard. The median SR was identical in both groups (6.0; interquartile range [IQR] of 4.7–7.7 vs. 5.5–6.3; *p* = 1.000). The ROC‐derived SR cutoff of 7.0 yielded an area under the curve of 0.500, indicating no discriminatory ability. The SR was not correlated with CT attenuation, the fibrosis status, or POPF. No adverse events occurred during EUS‐SE.

**Conclusion:**

In this pilot study, preoperative EUS‐SE did not predict intraoperative pancreatic textures. Strain‐based elastography alone appears to be insufficient for hardness stratification and should be complemented by more quantitative modalities.

**Abbreviations**
CI: confidence interval
CT: computed tomography
DP: distal pancreatectomy
EUS: endoscopic ultrasound
HU: Hounsfield unit
IQR: interquartile range
ISGPS: International Study Group on Pancreatic Surgery
NET‐G2: Grade‐2 Neuroendocrine Tumor
OR: odds ratio
POPF: postoperative pancreatic fistula
ROI: region of interest
SE: strain elastography
SPN: solid pseudopapillary neoplasm
SR: strain ratio
SSPPD: subtotal stomach‐preserving pancreaticoduodenectomy
SWE: shear‐wave elastography

## 1 Introduction

Postoperative pancreatic fistula (POPF) remains one of the most feared complications after a pancreatectomy, prolonging hospital stays and increasing the morbidity and mortality rates [1]. Numerous studies have identified soft pancreatic texture as an independent predictor of clinically relevant POPF, with two‐ to three‐fold reported odds ratios (ORs) relative to those of a firm gland [2, 3]. Accordingly, reliably assessing pancreatic hardness has important implications for perioperative decision‐making and patient counseling [4].

Currently, pancreatic textures are assessed primarily by surgeons performing palpation during surgery. Although this technique is subjective, several studies have demonstrated a strong correlation between intraoperative palpation and mechanical measurements obtained using a durometer, thereby supporting its use as a practical gold standard. For example, Hong et al. reported a Spearman correlation coefficient of 0.79 (*p* < 0.001) between a surgeon's tactile impressions and durometer‐derived hardness values in 78 pancreatectomy samples [5]. Nevertheless, palpation cannot be performed during robot‐assisted surgery, and no universally accepted quantitative preoperative method is available.

Czarnecka et al. recently proposed a preoperative soft pancreas risk score that combines clinical factors such as sex, the body mass index (BMI), and the pancreatic duct diameter [2]. Although it is helpful, this score does not directly measure parenchymal stiffness. In contrast, endoscopic ultrasound strain elastography (EUS‐SE) provides semiquantitative tissue hardness estimates. It has been proven useful for differentiating pancreatic tumors [6] and for grading fibrosis in chronic pancreatitis patients [7, 8]; however, its utility in nontumorous pancreatic tissues before surgery is performed remains largely unexplored, and no diagnostic cutoffs have been established for defining a “soft” or “hard” pancreas. Moreover, while EUS‐SE has been investigated in various pathological contexts, few studies have examined its ability to reflect intraoperative tactile assessments of pancreatic textures. To our knowledge, no previous reports have directly compared preoperative EUS‐derived strain values with the subjective classifications (soft vs. hard pancreases) of a surgeon at the time of surgery. In addition to EUS‐based approaches, computed tomography (CT) attenuation was measured as an indirect marker of pancreatic fat infiltration, given the lack of clarity regarding its association with pancreatic texture and POPF risk [9].

Therefore, we conducted a prospective pilot study to determine whether preoperative EUS‐SE could predict intraoperative pancreatic textures in patients undergoing pancreatectomies. By limiting all measurements to a single experienced endoscopist, we sought to minimize interobserver variability and isolate the intrinsic diagnostic performance of the technique.

## 2 Methods

### 2.1 Study Design and Patients

This single‐center prospective observational study was conducted at Toyonaka Municipal Hospital between October 2020 and March 2023. Consecutive patients scheduled for pancreatic resections who underwent preoperative EUS were screened. Only patients who provided written informed consent were enrolled in this study. Tumor staging was performed according to the eighth edition of the General Rules for the Study of Pancreatic Cancer issued by the Japan Pancreas Society [10]. The study was reviewed and approved by the Institutional Review Board of Toyonaka Municipal Hospital (approval no. 2020‐08‐10‐3) and conducted in accordance with the Declaration of Helsinki.

### 2.2 EUS Equipment and Elastography Procedure

**FIGURE 1:**
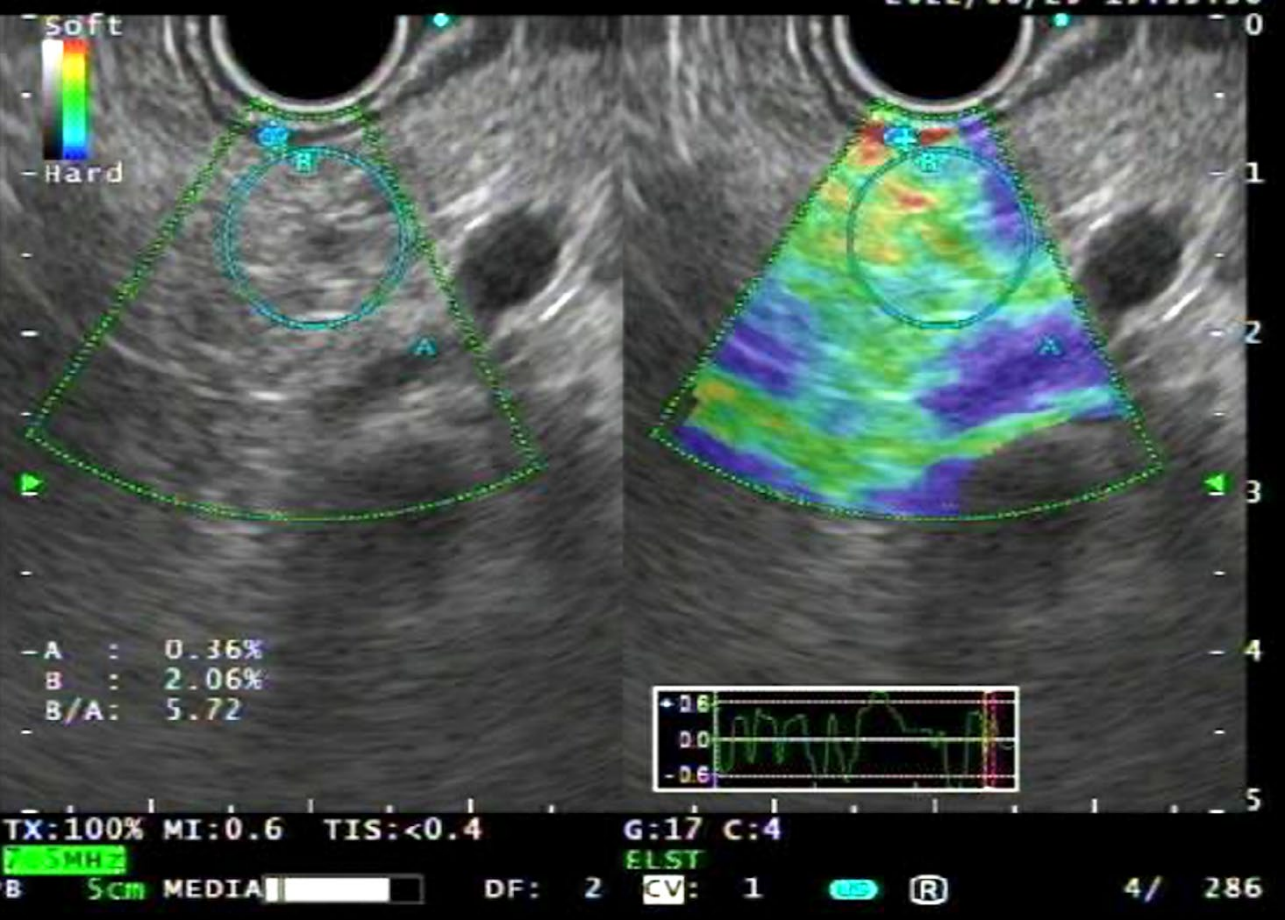
A circular ROI (A) was set within the nontumorous pancreatic parenchyma, and the strain value was 0.36%. A smaller ROI (B) was set in gastric wall tissue (shown in red in the right panel of the EUS image), and the strain value was 2.06%. The calculated strain ratio was 5.72.

EUS was performed using a GF‐UCT260 linear‐array echoendoscope and an EU‐ME2 PREMIER PLUS processor (Olympus Medical Systems, Tokyo, Japan). All examinations, including EUS‐SE, were performed by a single experienced endoscopist (K.M.) who had performed > 1000 EUS procedures before this study was initiated. EUS‐SE was conducted as part of the standard preoperative EUS‐fine needle aspiration (FNA) diagnosis procedure. For each patient, the strain ratio (SR) was defined as the ratio of the strain in the nontumorous pancreatic parenchyma (target region) to that in the gastric wall (reference region). A circular region of interest (ROI) of ≥ 5 mm was placed within the parenchyma, and a larger ROI was set in the gastric wall. Images were frozen once a stable elastographic pattern was maintained for ≥ 5 s, and the SR was calculated using ELST software (Olympus) (Figure 1). At least five measurements were obtained for each patient, and the mean SR was used for analysis purposes.

After the mid‐study transition to the Fujifilm SU‐9000 processor with the EG‐580UT linear‐array echoendoscope (Fujifilm, Tokyo, Japan), the opportunity to perform SE markedly decreased because this functionality was only available on the Olympus platform at our institution. Consequently, all SR measurements in this study were obtained exclusively with the Olympus EU‐ME2 PREMIER PLUS processor and GF‐UCT260 echoendoscope before the transition.

### 2.3 CT‐Based Evaluation of Pancreatic Tissue

Unenhanced CT images of the pancreas were acquired by either a Revolution GSI or Revolution EVO scanner (GE Healthcare Japan, Tokyo, Japan) and reviewed using the SYNAPSE VINCENT system (Fujifilm, Tokyo, Japan). ROIs were drawn on the largest cross‐section of the pancreas, excluding the tumor tissue and dilated ducts. All measurements were performed by a single investigator (T.N.).

In addition, prior to major manipulation, intraoperative palpation of the pancreas (soft/hard) was performed by a single attending surgeon (J.S.) who was blinded to EUS‐SE and CT results.

### 2.4 Study Endpoints

The primary endpoint was the correlation between the preoperative SR and the intraoperative pancreatic hardness status (soft vs. hard), as determined by the operating surgeon. A receiver operating characteristic (ROC) curve analysis was used to derive the SR cutoff.

The secondary endpoints included associations between the SR and CT attenuation values, histopathological fibrosis, and the occurrence of POPF. POPF was defined according to the International Study Group on Pancreatic Surgery (ISGPS) 2016 criteria [11].

### 2.5 Sample Size and Statistical Analysis

As a pilot study with exploratory intent, we pragmatically targeted a sample size of 20 patients on the basis of feasibility, and no formal power calculation was performed. A mid‐course feasibility check (not a hypothesis‐testing interim analysis) was planned after the first 10 patients were enrolled. However, owing to multiple factors, including the COVID‐19 pandemic, logistical constraints, and a mid‐study transition of our hospital's main EUS system from Olympus to Fujifilm, we were unable to continue enrollment using the same modality; to avoid cross‐platform heterogeneity, enrollment was stopped at nine consecutive eligible cases. All enrolled patients were included in the analyses. As SE was available only on the Olympus platform at our institution, we terminated accrual after nine consecutive eligible cases.

Continuous variables are reported as medians with interquartile ranges (IQRs) and compared via the Wilcoxon rank‐sum test, whereas categorical variables are presented as counts (percentages) and analyzed using the *χ* ^2^ test or Fisher's exact test (as appropriate). To identify preoperative predictors of pancreas softness, we performed univariate logistic regression with age, the SR, and unenhanced CT attenuation as covariates. All analyses were carried out using JMP Pro 17.2 (SAS Institute, Cary, NC, USA). The statistical significance level was set at *p* < 0.05.

## 3 Results

### 3.1 Patient Characteristics

Nine patients (median age: 69 years; IQR: 64.5–75.5; seven men) were enrolled (Table 1). Because no clinically meaningful correlation was observed during the interim analysis, the patient enrollment process was discontinued after nine cases. The tumor locations were the pancreatic head (*n* = 3), body (*n* = 1), and tail (*n* = 5). The surgical procedures included distal pancreatectomy (*n* = 6) and subtotal stomach‐preserving pancreaticoduodenectomy (SSPPD) (*n* = 3). The pathological diagnoses included adenocarcinoma (*n* = 7), a Grade‐2 Neuroendocrine Tumor (*n* = 1), and a solid pseudopapillary neoplasm (*n* = 1). All patients with adenocarcinomas received neoadjuvant chemotherapy. Histopathological nontumorous tissue fibrosis was present in four patients (44%).

**TABLE 1:** Clinical background of the patients.

Case	Age	Sex	Tumor location	Hardness according to palpation	Average SR values	Average CT (HU)	cT	cN	cM	cStage	Neoadjuvant chemotherapy	Surgical procedure	Pathological diagnosis	Presence of fibrosis based on pathological findings
1	72	M	Tail	Hard	5.5	37.8	T3	N1	M0	IIB	Yes	DP	Adenocarcinoma	Yes
2	63	M	Head	Hard	6.6	39.2	T3	N0	M0	IIA	Yes	SSPPD	Adenocarcinoma	No
3	71	M	Tail	Hard	6.0	20.9	T3	N0	M0	IIA	Yes	DP	Adenocarcinoma	Yes
4	46	F	Tail	Soft	7.4	43.7	NA	NA	NA	NA	No	DP	SPN	No
5	69	M	Body	Soft	4.7	33.4	T1	N0	M0	IA	No	DP	NET‐G2	No
6	66	M	Tail	Soft	8.7	11.4	T3	N0	M0	IIA	Yes	DP	Adenocarcinoma	Yes
7	79	F	Tail	Soft	5.1	20.8	T3	N0	M0	IIA	Yes	DP	Adenocarcinoma	No
8	80	M	Head	Soft	7.0	34	T3	N0	M0	IIA	Yes	SSPPD	Adenocarcinoma	No
9	68	M	Head	Soft	4.7	39.4	T1	N0	M0	IA	Yes	SSPPD	Adenocarcinoma	Yes

Abbreviations: DP, distal pancreatectomy; NA, not applicable; NET‐G2, Grade‐2 neuroendocrine tumor; SPN, solid pseudopapillary neoplasm; SR, strain ratio; SSPPD, subtotal stomach‐preserving pancreatoduodenectomy.

EUS‐SE was technically successful in all patients, with no adverse procedure‐related events and no cases of clinically relevant POPF.

### 3.2 Pancreatic Hardness and EUS‐SE Findings

**FIGURE 2:**
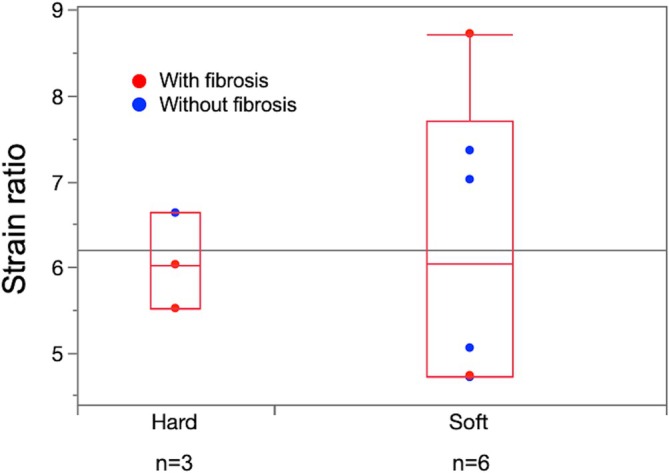
Comparison between the SR values of soft pancreases (*n* = 6) and hard pancreases (*n* = 3) as assessed via intraoperative palpation. Each dot represents an individual patient. The red circles indicate patients with histopathological fibrosis in their nontumorous pancreatic parenchyma.

Through intraoperative palpation, the pancreases were categorized as soft in six patients (67%) and as hard in three patients (33%). The median SRs of the soft and hard groups were identical (6.0; IQR: 4.7–7.7 vs. 5.5–6.3; *p* = 1.000 [Figure 2]) (Table 2). An ROC analysis yielded an SR cutoff of 7.0 with an area under the curve of 0.500, indicating no discriminatory ability.

**TABLE 2:** Comparison between the clinical backgrounds of patients with soft and hard pancreases.

	Soft pancreas	Hard pancreas	*p*
*n*	6	3	
Strain ratio (IQR)	6.0 (4.7–7.7)	6.0 (5.5–6.3)	1.0000
Sex, male *n* (%)	4 (67)	3 (100)	0.5000
Age	68.8 (61–79)	71 (63–72)	1.0000
CT value (HU)	33.7 (18.5, 40.5)	37.8 (20.9, 39.2)	0.8973
*Tumor location*			
Head/body/tail	2/1/3	1/0/2	0.7408
Pathological diagnosis, adenocarcinoma	4 (67)	3 (100)	0.5258
Presence of fibrosis based on pathological findings	2 (33)	2 (67)	0.5238

Abbreviation: HU, Hounsfield unit.

### 3.3 CT Attenuation and Pancreatic Texture

The median CT attenuation of the pancreatic parenchyma was 34 HU (IQR: 20.9–39.3). Although the CT values tended to be lower in the soft pancreas group (33.7 HU) than in the hard pancreas group (37.8 HU), this difference was not significant (*p* = 0.897) (Table 2).

### 3.4 Preoperative Predictors of Pancreas Softness

Univariate logistic regression revealed no significant associations between pancreas softness and age (OR: 0.77; 95% confidence interval [CI]: 0.005–122; *p* = 0.92), SR (OR: 1.68; 95% CI: 0.02–143; *p* = 0.82), or CT attenuation (OR: 0.98; 95% CI: 0.85–1.13; *p* = 0.76).

## 4 Discussion

Our prospective pilot study investigated whether preoperative EUS‐SE could intraoperatively discriminate between soft and hard pancreases. Despite the intuitive appeal of this technique, EUS‐SE directly measures parenchymal stiffness rather than relying on indirect clinical surrogates. However, no discriminative signal emerged in nine consecutive patients; the median SRs of the soft and hard groups were identical, and the area under the ROC curve was 0.500. Accordingly, future validation should involve multicenter and prospective studies rather than rely on larger samples alone. As EUS‐SE directly measures actual hardness, the fact that this tightly controlled, single‐operator pilot study failed to discriminate between soft and hard pancreases suggests that the true difference between them is very small. Therefore, we conclude that simply increasing the sample size might result in statistical significance, but there is a high probability that doing so will not result in a clinically significant difference.

Previous studies using EUS‐SE have demonstrated correlations between SR and histological fibrosis. Itoh et al. reported a correlation coefficient of 0.68 between SR and the fibrosis score in resected pancreatic specimens, with an SR ≥ 2.25 predicting severe fibrosis [12]. In a narrative review, Yamamiya et al. concluded that SE can quantitatively assess pancreatic stiffness and correlate with histology, while noting limitations such as heterogeneous fibrosis distribution and operator‐dependent variability [8]. A meta‐analysis by Mei et al. found that SE has high pooled sensitivity (95%) but moderate specificity (67%) for differentiating solid pancreatic masses, supporting its role as a complementary rather than a standalone diagnostic tool [6]. Because these investigations employed the same strain‐based measurement principle as our study, their findings can be interpreted in a directly comparable context. All of these studies used SE rather than shear wave elastography (SWE), making them directly comparable to our methodology.

EUS‐SE is a noninvasive imaging modality that has demonstrated strong diagnostic performance in terms of assessing liver fibrosis [13]. Its application for treating pancreatic diseases, particularly for evaluating fibrosis in chronic pancreatitis, has also been reported [7, 8]. However, in this study, which focused on nontumorous pancreatic parenchyma, EUS‐SE did not effectively reflect the textures assessed during surgery.

One possible explanation for this outcome is the technical limitations of SE. This technique visualizes the tissue deformation process under external pressure and provides relative rather than absolute stiffness values and can therefore be affected by probe pressure levels, patient positioning statuses, operator techniques, and surrounding tissue properties, compromising reproducibility [14]. In contrast, SWE measures the propagation speeds of shear waves and yields absolute stiffness values in kilopascals (kPa), making it less operator‐dependent and more reproducible [15, 16, 17]. Several comparative studies have demonstrated the superior reproducibility, lower operator dependency, and better interobserver agreement of SWE compared with SE [16, 17]. SWE has quantitative advantages, particularly in superficial tissues [18], and, unlike SE, its performance is less influenced by operator‐dependent factors. Although shear‐wave attenuation can reduce accuracy for deep targets, the short distance between the gastrointestinal tract and pancreas in EUS largely mitigates this limitation, allowing SWE to retain its quantitative advantage [18]. Although the use of EUS‐guided SWE remains limited due to equipment availability issues, it demonstrates considerable promise for future standardized pancreatic assessments [14, 19].

In addition to technical issues, clinical and procedural factors may also have influenced the difference between the findings of EUS‐SE and intraoperative palpation. Most patients in our cohort received neoadjuvant chemotherapy, which may have induced fibrosis, edema, or inflammation that altered their intraoperative textures. However, EUS‐SE is often performed before chemotherapy, potentially leading to inconsistencies between preoperative imaging and surgical findings. A previous study demonstrated that increased pancreatic stiffness was observed when using a durometer due to a single dose of preoperative radiotherapy [20]; however, no clear evidence currently supports the presence of similar effects for chemotherapy alone. Therefore, the effect of chemotherapy alone on pancreatic stiffness remains uncertain and warrants further investigation.

This study had several limitations. First, the sample size (*n* = 9) was small, as enrollment was intentionally discontinued after a planned mid‐course feasibility check demonstrated no clear trends or discriminatory ability of EUS‐SE for pancreatic hardness. Consequently, the effect estimates had wide CIs, and the generalizability of the results may be limited by device‐specific implementation because of selection, measurement bias, and lack of blinding. The original target sample size was set at 20 based on the feasibility of a pilot study; however, multiple factors contributed to the early termination. These included logistical constraints during the COVID‐19 pandemic, a mid‐study transition of our hospital's main EUS system (from Olympus to Fujifilm), and—most importantly—the interim analysis indicating that further enrollment was unlikely to change the negative result. We judged that simply increasing the sample size would not improve clinical utility, given the lack of even a weak association in the initial cohort. Second, this study was conducted at a single center, and all procedures were performed by a single experienced operator, which may have limited its generalizability. Finally, the platform transition and resulting software incompatibility further limited enrollment and prevented SR analyses in later cases. Future studies should incorporate quantitative SWE to establish reproducible cutoffs and achieve enhanced clinical applicability. Additionally, ROI placement may vary slightly between operators and even within the same operator, potentially introducing measurement variability. To minimize this variability, all procedures were performed by a single experienced endoscopist. Furthermore, because SE provides relative rather than absolute stiffness values, factors such as ROI size, surrounding tissue characteristics, and probe pressure may have influenced the measurements despite standardization efforts. Finally, no clinically relevant POPF occurred in this cohort, which precluded assessment of the relationship between preoperative EUS‐SE parameters and POPF risk. This pilot study did not demonstrate that the SR showed diagnostic utility for the preoperative prediction of pancreatic texture. In addition, subsequent studies will adopt a prespecified sample size powered to detect clinically meaningful discrimination while accounting for the expected prevalence of a soft gland.

## 5 Conclusion

In this pilot study, the preoperative pancreatic stiffness measured by EUS‐SE was not significantly correlated with intraoperative pancreatic textures. Although EUS‐SE is a noninvasive and convenient method, our findings highlight its important technical limitations, including operator dependence and limited reproducibility. Future studies should incorporate SWE to conduct more objective and quantitative assessments and evaluate the impacts of clinical factors, such as neoadjuvant chemotherapy.

## Conflicts of Interest

The authors declare no conflicts of interest.

## Data Availability

The data that support the findings of this study are available on request from the corresponding author. The data are not publicly available due to privacy or ethical restrictions.
